# Click chemistry-based tracking reveals putative cell wall-located auxin binding sites in expanding cells

**DOI:** 10.1038/s41598-017-16281-w

**Published:** 2017-11-22

**Authors:** Jozef Mravec, Stjepan K. Kračun, Elena Zemlyanskaya, Maja G. Rydahl, Xiaoyuan Guo, Martina Pičmanová, Kasper K. Sørensen, Kamil Růžička, William G. T. Willats

**Affiliations:** 1 Department of Plant and Environmental Sciences, University of Copenhagen, Thorvaldsensvej 40, 1871 Frederiksberg-C, Denmark; 2 CEITEC Masaryk University, Kamenice 5, CZ-625 00 Brno, Czechia; 3 Department of Chemistry, University of Copenhagen, Thorvaldsensvej 40, 1871 Frederiksberg-C, Denmark; 4 Laboratory of Hormonal Regulations in Plants, Institute of Experimental Botany, Czech Academy of Sciences, Rozvojová 263, CZ-165 02 Prague, Czechia; 5 School of Agriculture, Food and Rural Development, Newcastle University, Newcastle upon Tyne, NE1 7RU UK

## Abstract

Auxin is a key plant regulatory molecule, which acts upon a plethora of cellular processes, including those related to cell differentiation and elongation. Despite the stunning progress in all disciplines of auxin research, the mechanisms of auxin-mediated rapid promotion of cell expansion and underlying rearrangement of cell wall components are poorly understood. This is partly due to the limitations of current methodologies for probing auxin. Here we describe a click chemistry-based approach, using an azido derivative of indole-3-propionic acid. This compound is as an active auxin analogue, which can be tagged *in situ*. Using this new tool, we demonstrate the existence of putative auxin binding sites in the cell walls of expanding/elongating cells. These binding sites are of protein nature but are distinct from those provided by the extensively studied AUXIN BINDING PROTEIN 1 (ABP1). Using immunohistochemistry, we have shown the apoplastic presence of endogenous auxin epitopes recognised by an anti-IAA antibody. Our results are intriguingly in line with previous observations suggesting some transcription-independent (non-genomic) activity of auxin in cell elongation.

## Introduction

Plant hormone auxin regulates various aspects of plant development and growth^1^. Auxin synthesis, transport, turnover and signalling is mediated by compartmentalized pathways and trafficked protein modules^2–6^. Much effort has been devoted to the study of the dynamic auxin distribution within plant organs and tissues required for patterning processes or tropic responses^3,4^. However, the precise subcellular localization of auxin is poorly described, which limits our understanding of some of the mechanisms of auxin regulation. This is especially true for the effect of auxin in the promotion of rapid expansion. The so-called acid growth theory proposes that auxin activates the plasma membrane H^+^-ATPase and the resulting pH change is conveyed to expansion-facilitated cell wall loosening^7–9^. has been suggested over the years that this mechanism is transcription-independent (or so called ‘non-genomic’)^10,11^. However, some recent publications revisited the nuclear signalling dependence of this process and demonstrated the important role of auxin-induced SAUR proteins^12–14^ in hypocotyl elongation. On the other hand, the extremely rapid effect of auxin in root elongation within a timeframe of minutes, observed already in the early 1990s^15^, strongly suggests that some non-genomic direct activity of auxin is taking place. To study such complex and elusive biological phenomena, efficient and reliable tools for auxin detection are of a great importance.

Immunolocalization using anti-auxin antibodies and its recently reported fluorescently-labelled analogues can be used to visualize auxin accumulation sites^16–19^, but these methods have some drawbacks. The small auxin epitope recognized by antibodies limits their specificity and may not be accessible when IAA is bound to proteins. In the case of fluorescent auxins, the bulky fluorescent tag increases the risk of biased targeting and/or metabolic processing.

The problems of these approaches have been partly overcome for some molecules in animal models by ‘click chemistry’^20,21^. Instead of using large *in vitro*-generated fluorescent conjugates, this technology is based on a highly selective reaction triggered *in situ*. The most common reaction is Huisgen dipolar 1,3-cycloaddition^20^ between an alkyne and an azide, one of the groups being present on the molecule of interest and the other on a detection tag. Both groups are relatively inert, normally not present in living organisms, and usually do not tamper with the function of the tagged molecule. This method also enables access to a high variety of tags which can be used for labelling: fluorescent, enzymatic or nanogold particles for electron microscopy. Unfortunately, only few examples of the application of click chemistry have been reported in plants, dealing mainly with studies of incorporation of modified monosaccharides into cell wall components^22^.

**Figure 1:**
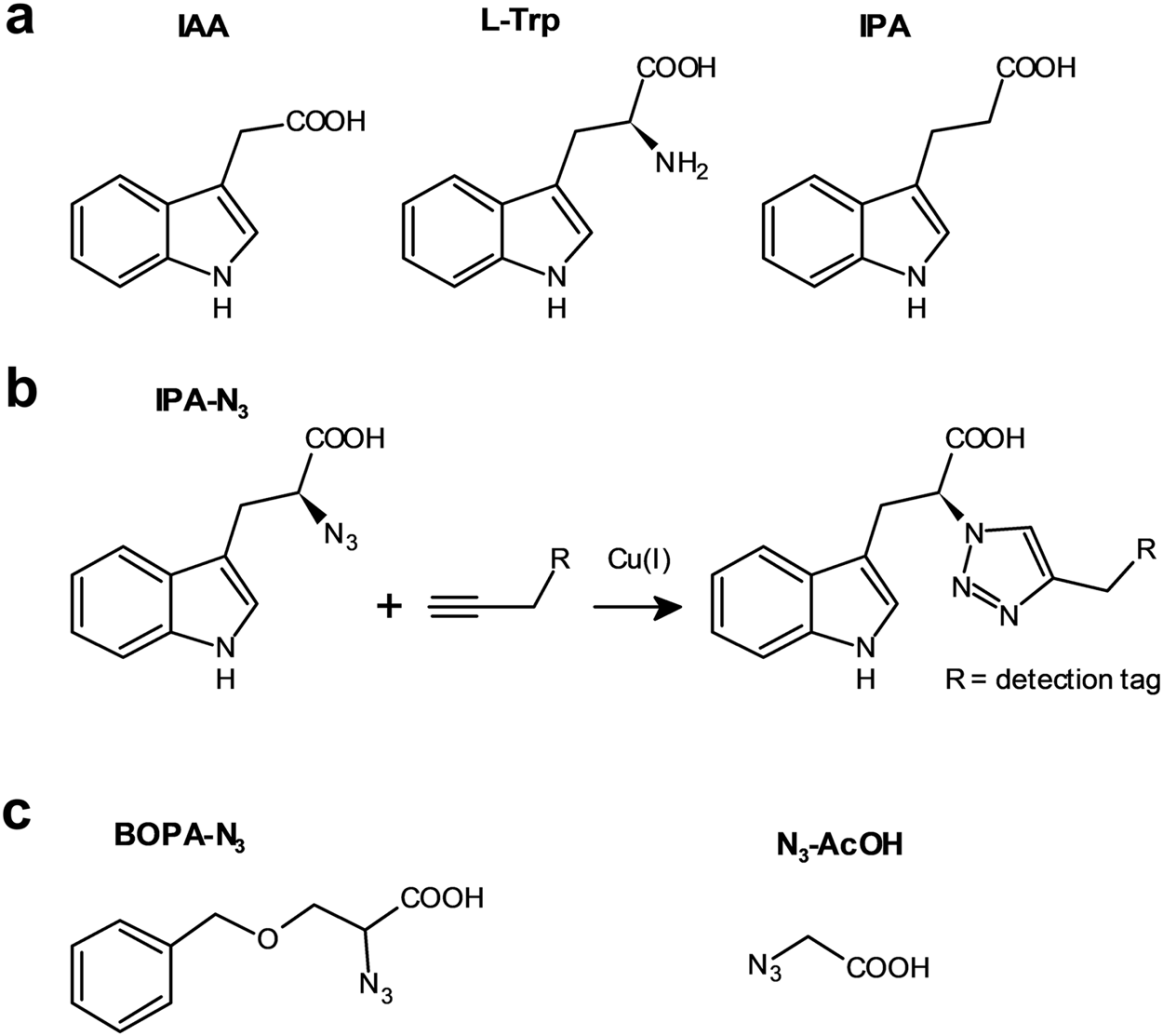
Molecular structures of the relevant compounds. (**a**) The molecular structures of IAA, L-tryptophan, IPA and (**b**) IPA-N_3_. Note the differences in the C2 carbon. (**b**) IPA-N_3_ can be reacted to an alkyne-functionalized tag via Huisgen dipolar 1,3-cycloaddition. (**c**) Structure of the used control compounds bearing azido and carboxy group: 2-azido-3-benzyloxypropionic acid (BOPA-N_3_) and azido acetic acid (N_3_-AcOH).

Our strategy was to explore this technology for auxin localisation using a click chemistry-ready auxin analogue. Aryl azido-IAA derivatives have been used in the past to identify auxin binding proteins^23–25^; however, they are photosensitive^26^. Indole-3-propionic acid (IPA) has been identified as occurring in some plant species^27,28^, and to some extent shows IAA-like properties in transport and signalling^29^. Its azido derivative (*S*)-2-azido-3-(3-indolyl)propionic acid (IPA-N_3_; Fig. 1) is a commercially available click chemistry reagent, intended to study the dynamics of alkaloid receptors in animal cells. We explored the possibility of using IPA-N_3_ as an auxin tracer and provided some evidence of the presence of auxin binding sites in cell walls of elongating cells.

## Results

### IPA-N_3_ shows similar activity to IAA in bioassays

**Figure 2:**
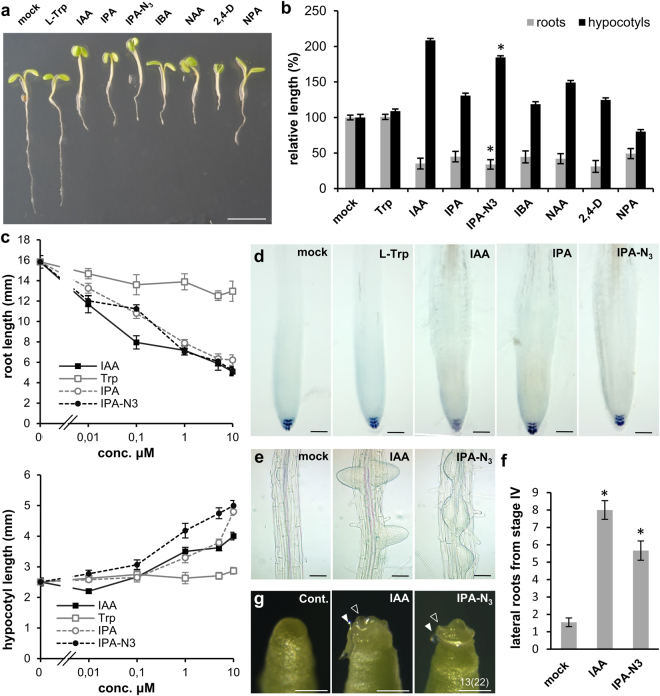
Characterization of the IPA-N_3_ auxin activity. (**a**,**b**) Comparison of the phenotypes exerted by various auxin-related compounds. (**a**) Seedlings grown for 48 h in the liquid culture supplemented with various compounds (5 μM concentration). Application of IAA, IPA and IPA-N_3_, but not L-tryptophan induced identical phenotypes. Note the epinastic cotyledons, enhanced hypocotyl but inhibited root elongation. These phenotypes were less pronounced in the case of the precursor of IBA and synthetic auxin NAA. The non-transportable synthetic auxin 2,4-D caused severe retardation of growth, and the auxin transport inhibitor NPA showed dissimilar phenotypes – e.g. cotyledons did not open. (**b**) Quantification of the root and hypocotyl length of above seedlings. (**c**) Sensitivity test shows similar activity range for the four related auxinic compounds tested. The mean values of at least 12 measurements ± SE. The difference in root length between mock and 10 nM IPA-N_3_ is statistically significant. *p < 0.01 (Student’s *t*-test). (**d**) Morphology of root tips of seedlings from (**b**). Note the root tip swelling in the seedlings grown in the three auxinic compounds indicated. (**e**–**g**) Two assays indicating that IPA-N_3_ exhibits morphogenic-like activity. (**e**,**f**) IPA-N_3_ is able to induce ectopic lateral root initiation. (**e**) Depiction of lateral root initiation in mock, IPA-N_3_ and IAA treated plants (5 µM, 48 h) (**f**) Quantification of the effect. Number of lateral root initiates from stage IV. The mean values from at least 9 roots ± SE. *p < 0.01 (Student’s *t*-test). (**g**) IPA-N_3_ triggers organ formation on the *pin1* shoot meristem. The position of the application of the lanolin paste is indicated by a closed arrowhead and the outgrowth of an organ by an open arrowhead. The experiment was repeated twice on at least 10 plants for each treatment. The number of the positive cases of outhgrowth from all samples in total are indicated. Scale bars = 5 mm (**a**), 50 μm (**d**,**e**), 1 mm (**g**).

First, we tested whether IPA-N_3_ has cellular activity comparable to the major form of auxin (IAA) and compared it to other auxinic compounds. We were particularly interested in IPA and L-tryptophan, which differ from IPA-N_3_ based on the substituent on the carbon C2 (Fig. 1a). We also included four other auxin-related compounds in our analyses: a precursor of IAA, indole-butyric-acid (IBA)^2^, synthetic auxins: 1-naphthalene acetic acid (NAA) and 2,4-dichlorophenoxyacetic acid (2,4-D) and an auxin transport inhibitor *N*-(1-naphthyl)phthalamidic acid (NPA)^29,30^. Incubation of *Arabidopsis* seedlings in 5 μM IAA, IPA, as well as IPA-N_3_, but not L-tryptophan induced comparable phenotypes in *Arabidopsis* seedlings, including highly elongated hypocotyls, inhibited root elongation and epinastic cotyledons. Interestingly, the effects of IPA-N_3_ resembled IAA more than any other auxinic compound tested (Fig. 2a,b), including a response to IPA-N_3_ already at a 10 nM concentration in a root elongation assay (Fig. 2c). At the microscopic level, IPA-N_3_ treated seedlings also showed swelled root tip appearance (Fig. 2d) and ectopic lateral root initiation as IAA (Fig. 2e,f). The morphogen activity of IPA-N_3_ could also be demonstrated by inducing organ initiation on the shoot meristem of the *pin1* mutant (Fig. 2g) defective in forming auxin maxima^31,32^. This shows that substitution with an azido group on C2 is not inhibitory for its activity and IPA-N_3_ can be considered an auxinic analogue.

### IPA-N_3_ activates auxin-dependent transcriptional response

**Figure 3:**
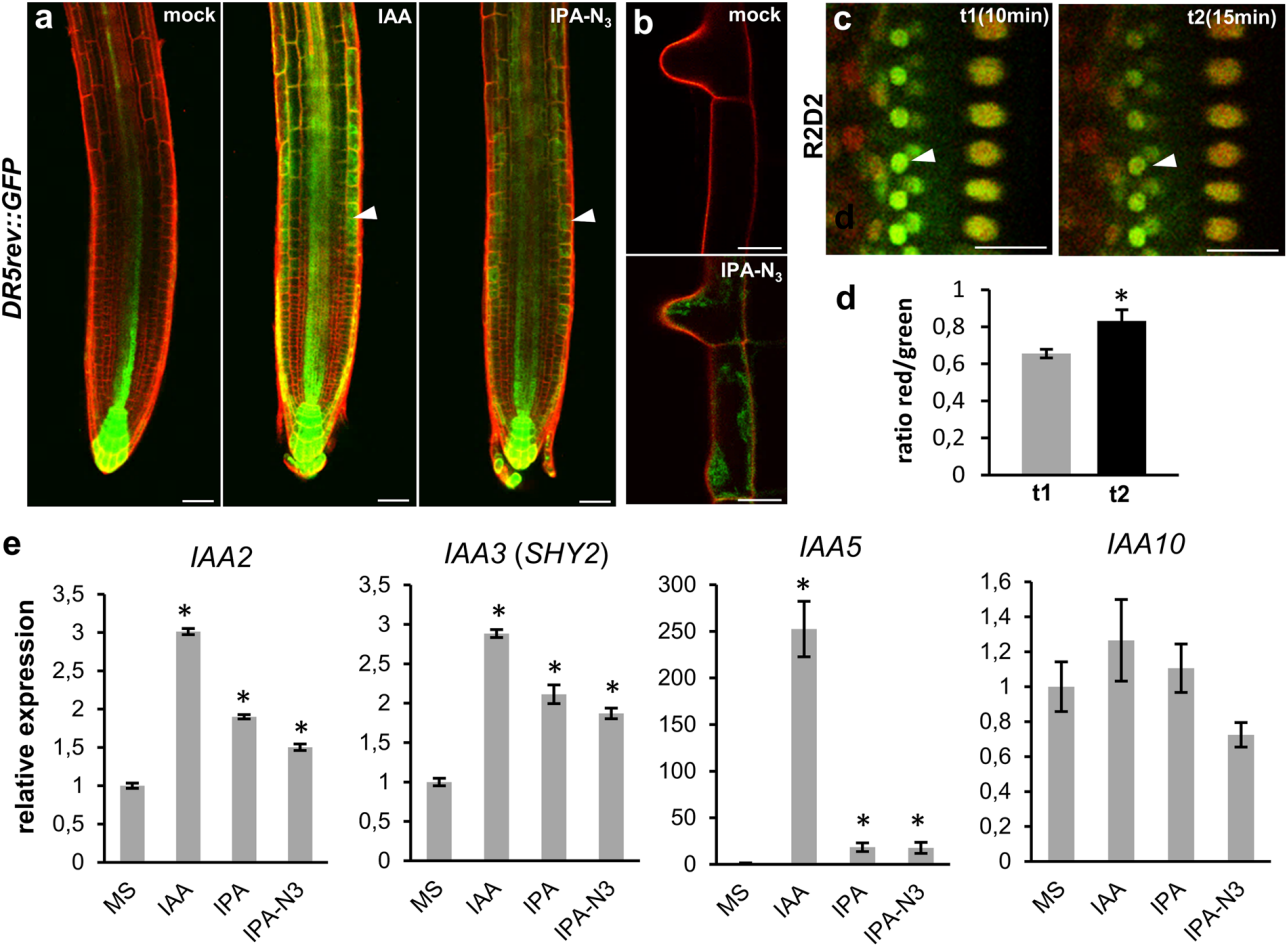
Activation of auxin transcriptional reporter by IAA and IPA-N_3_. (**a**,**b**) The induction of *DR5rev::GFP* reporter in the root tip (**a**), and trichoblasts (**b**), could be observed 4 h after the IAA and IPA-N_3_ application (5 μM). Note the accumulation of the ER-targeted GFP expressed from the auxin inducible *DR5rev* promoter in epidermal cells (arrowheads). Propidium iodide was used as counterstain to outline cells (red channel). (**c**) Sensitive auxin-induced degradation domain-based R2D2 reporter scanned at t1 = 10 min after IPA-N_3_ application (5 μM) and t2 = 15 min after application. Note the disappearance of the DII: n3Venus signal (arrowheads). (**d**) Quantification of the ratio between the red signal (mDII: ntdTomato; auxin insensitive) and green signal (DII: n3Venus; auxin sensitive) at time points t1 and t2. Mean of measurement from 5 cells ± SE. *p < 0.05 (Student’s *t*-test). (**e**) Quantitative RT-PCR on four auxin-regulated genes. Three tested genes were significantly induced after 1 h incubation with 1 μM of IPA or IPA-N_3_ potency with less than 1 μM IAA. The mean values of at least 7 measurements ± SE. *p < 0.01 (Student’s *t*-test). Scale bars = 20 μm (**a**), 10 μm (**b**,**c**).

We further examined the ability of IPA-N_3_ to activate auxin responsive reporter *DR5rev::GFP* ^16^. The transcriptional activation of *DR5rev* promoter and resulting GFP signal accumulation in the root tip and root hairs occurred after 4 h of IPA-N_3_ application, although with lesser intensity compared with IAA at the same concentration level (Fig. 3a,b). A more sensitive R2D2 auxin reporter line, which is based on early steps of auxin signalling dependent degradation of Aux/IAA^33^, was further used to assess the velocity of IPA-N_3_ activity. Compared to IAA, the response was weaker (data not shown); however, measurable degradation was observed within the timeframe of 15 min after IPA-N_3_ application (Fig. 3c,d). To further evaluate these reporter-based *in situ* observations, we performed qRT-PCR analysis on auxin-inducible genes. From four IAA genes, three were significantly induced with 1 µM of the compounds within 1 h after application. Again, IAA was the most potent among the tested compounds.

One of the possible explanations for the observed phenotypes, the activation of auxin reporters and induction of IAA genes, would be that the IPA-N_3_ standard is impure or that IPA-N_3_ is rapidly catabolised to IAA. To test this, we performed LC-MS analyses and confirmed that IPA-N_3_ preparation does not contain any traceable amount of IAA (Supplementary Fig. S1a). Further, we performed *in vitro* and *in vivo* tests for the rate of IPA-N_3_ catabolism using pea homogenates, as well as intact epicotyls (Supplementary Fig. S1b,c). The results pointed to the stability of IPA-N_3_ in the performed assays. Although we do not rule out some level of metabolic processing, given the velocity of induction of IAA genes or response of the R2D2 reporter and especially the root sensitivity to as low as 10 nM IPA-N_3,_ our experiments indicate that IPA-N_3_ can directly trigger SCF(TIR1/AFB)-mediated signalling.

### Detection of IPA-N_3_ in *Arabidopsis* root tip

**Figure 4:**
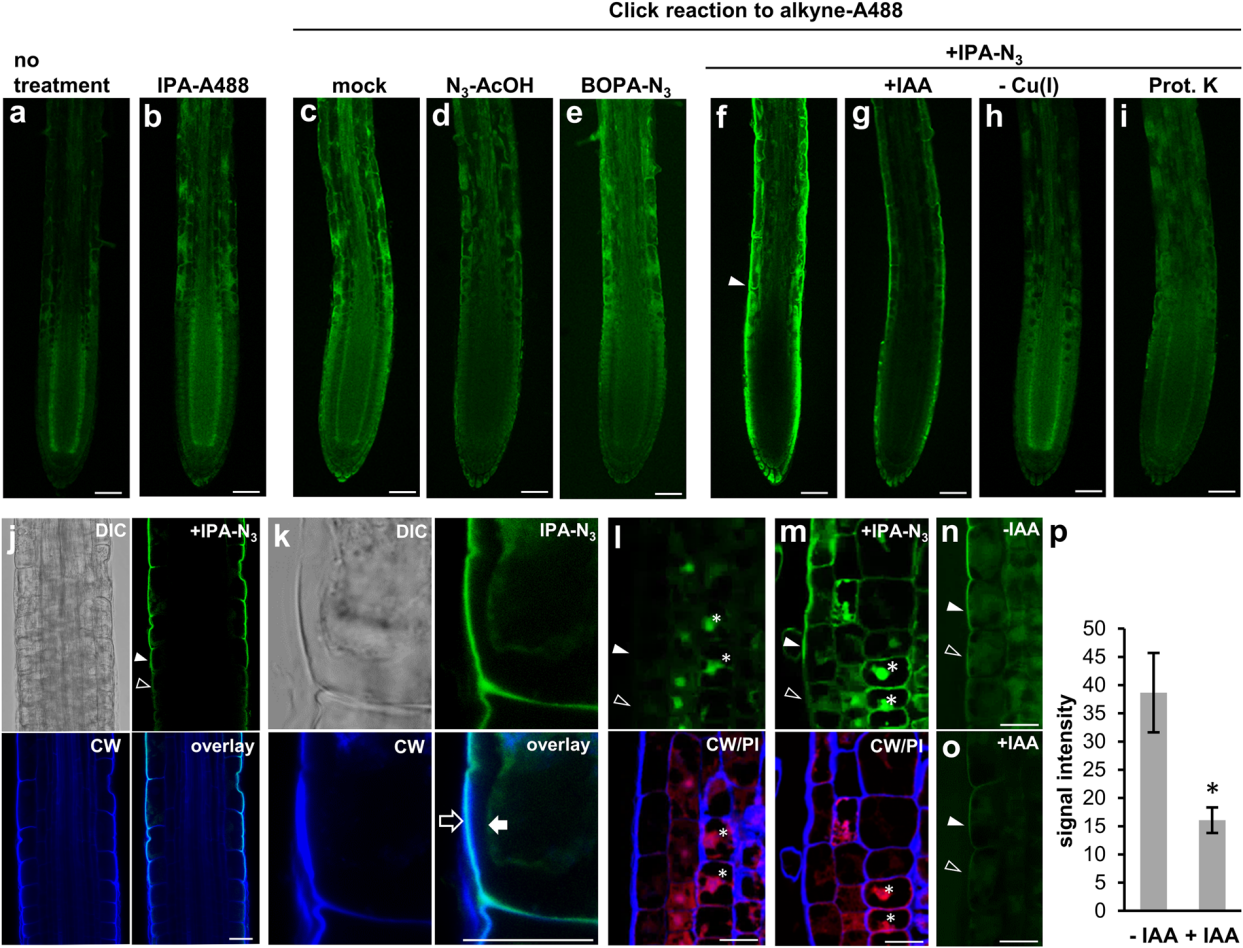
Localization of IPA-N_3_ in the *Arabidopsis* root tip using click chemistry. Detection of IPA-N_3_ in *Arabidopsis* roots after 1 h incubation (10 μM concentration of the compounds) followed by *in vivo* coupling to the alkyne functionalized fluorophore Alexa Fluor 488 in comparison with a set of controls scanned using LSCM with the same settings. (**a**) Fixed and non-treated seedlings, (**b**) seedlings incubated with *in vitro* conjugate of IPA-N_3_ to alkyne Alexa Fluor 488, (**c**–**i**) seedlings subjected to a click reaction after incubation in (**c**) mock, (**d**,**e**) control azido group-containing compounds (**d**) 2-azido acetic acid and (**e**) 2-azido-3-benzyloxypropionic acid. (**f**–**i**) Seedlings incubated with IPA-N_3_. (**f**) Seedlings incubated with IPA-N_3_ only. Note the signal in epidermis (arrowhead) and outer cell layer of root cap. (**g**) IPA-N_3_ signal can be reduced when seedlings are co-incubated with IAA (10 μM). (**h**) Absence of IPA-N_3_ signal can be observed when the copper catalyst of the reaction is omitted or (**i**) when the seedlings are pre-treated with the proteinase K. (**j**–**o**) Analyses of the IPA-N_3_ signal in the transition zone of the *Arabidopsis* roots. Cells before elongation are marked with open arrowheads and those elongating are marked with a closed arrowhead. (**j**) Scan of the transition zone with the lower photo multiplier (PMT) intensity as in (**f**) reveals that the majority of the IPA-N_3_ signal appears in the root epidermal cells starting to elongate. (**k**) Plasmolysis and co-localisation with cell wall marker Calcofluor White (CW) confirms the cell wall signal of IPA-N_3._ An open arrow marks the cell wall and a closed arrow marks the cytoplasm. (**l**,**m**) Analysis of IPA-N_3_ signal in semi-thin resin sections. Click labelling of the resin sections of the root tip in the area of the elongation zone of seedlings (upper panels) treated with (**l**) mock and (**m**) IPA-N_3_. Bottom panels: counterstain with propidium iodide marking nuclei (PI; red channel) and Calcofluor White marking cell walls (CW; blue channel). The position of the nuclei is marked with asterisks. Note the cell wall signal in epidermal cells and the enhanced nuclear signal in the IPA-N_3_ treated sample. (**n**,**o**) The effect of IAA on IPA-N_3_ binding in the transition zone of *Arabidopsis* roots. (**n**) Seedlings incubated with IPA-N_3_ only and (**o**) seedlings co-incubated with 10 μM IAA. Note the reduction of the signal in (**o**). (**p**) Quantification of the cell wall signal from both treatments. The reduction of the IPA-N_3_ signal by co-incubating with IAA has been observed in three independent experiments. The mean values of 7 measurements ± SE. *p < 0.01 (Student’s *t*-test). Scale bars = 20 μm (**a**–**i**), 10 μm (**j**–**o**).

Next, we directly visualized IPA-N_3_ *in situ* in *Arabidopsis* seedling root tips using click labelling. Three-day-old seedlings were first incubated for 1 hour in media containing 10 μM IPA-N_3_, washed and fixed with EDAC (1-ethyl-3-(3-dimethylaminopropyl)carbodiimide), which is able to form amide bonds from the carboxyl group of IPA-N_3_ and amino groups of nearby proteins. The seedlings were then subjected to a copper-catalysed cycloaddition and to an alkyne functionalized Alexa Fluor 488 dye, and we compared the labelling obtained with a set of controls (Fig. 4a–i). These included: (i) seedlings fixed but not further processed; incubated with: (ii) mock, (iii) with control compounds 2-azidoacetic acid and 2-azido-3-benzyloxy propionic acid (N_3_-AcOH, BOPA-N_3_; Fig. 1c) or (iv) with the fluorophore pre-reacted *in vitro* with IPA-N_3_. Further, we used controls involving (v) IPA-N_3_ incubation but omitting the copper catalyst in the reaction mixture, (vi) pre-incubating the seedlings with IAA (10 μM) or (vii) pre-treated with proteinase K.

Compared to these controls, a distinguishable IPA-N_3_ signal could be observed in the epidermis and outer layer of the root cap (Fig. 4f). However, the intracellular signal was not as well defined, and it was difficult to assess due to high levels of non-specific signals in the controls. The strongest signal from ‘clicked’ IPA-N_3_ could be observed in the root elongation zone. Closer examination of the transition zone showed that there is a clear specific appearance of the signal in the cell walls of cells at the onset of their elongation (Fig. 4j).

The presence of IPA-N_3_-derived fluorescence at the cell wall was confirmed by co-staining with the cell wall marker Calcofluor White^34^ and by cell plasmolysis (Fig. 4k). However, only the outer portion of cell walls was labelled (Fig. 4j,k). To address penetration issues we also performed resin embedding and sectioning of IPA-N_3_ treated root tips following the click reaction on the sections (Fig. 4l,m). The cell wall signal in elongating cells was again detectable mainly in the outer layer of epidermal cell walls, suggesting limited penetration of any reagents used before the click reaction.

More importantly, the cell wall signal could be reduced by co-incubating the seedlings with 10 μM IAA (Fig. 4n–p). These results suggested that although detection of IPA-N_3_ in *Arabidopsis* seedlings has apparent limitations for deeper layers of root tissues, it can generate specific labelling of cell walls in epidermal cells, specifically those undergoing elongation. They also hinted that IPA-N_3_ affinity for cell walls is mediated by protein(s), which is(are) also able to bind native IAA.

### Shared binding sites of IAA and IPA-N_3_ in root hair cell walls

During labelling experiments, we noticed that cell walls of root hairs are root structures most clearly labelled by IPA-N_3_. Root hairs are an interesting model to study auxin localization because their growth is regulated by auxin^35,36^ and it involves dynamic cell wall remodelling^37^. Moreover, they are not directly connected with any other cells, so the auxin cell wall localization is unlikely to be a result of cell-to-cell polar auxin transport.

**Figure 5:**
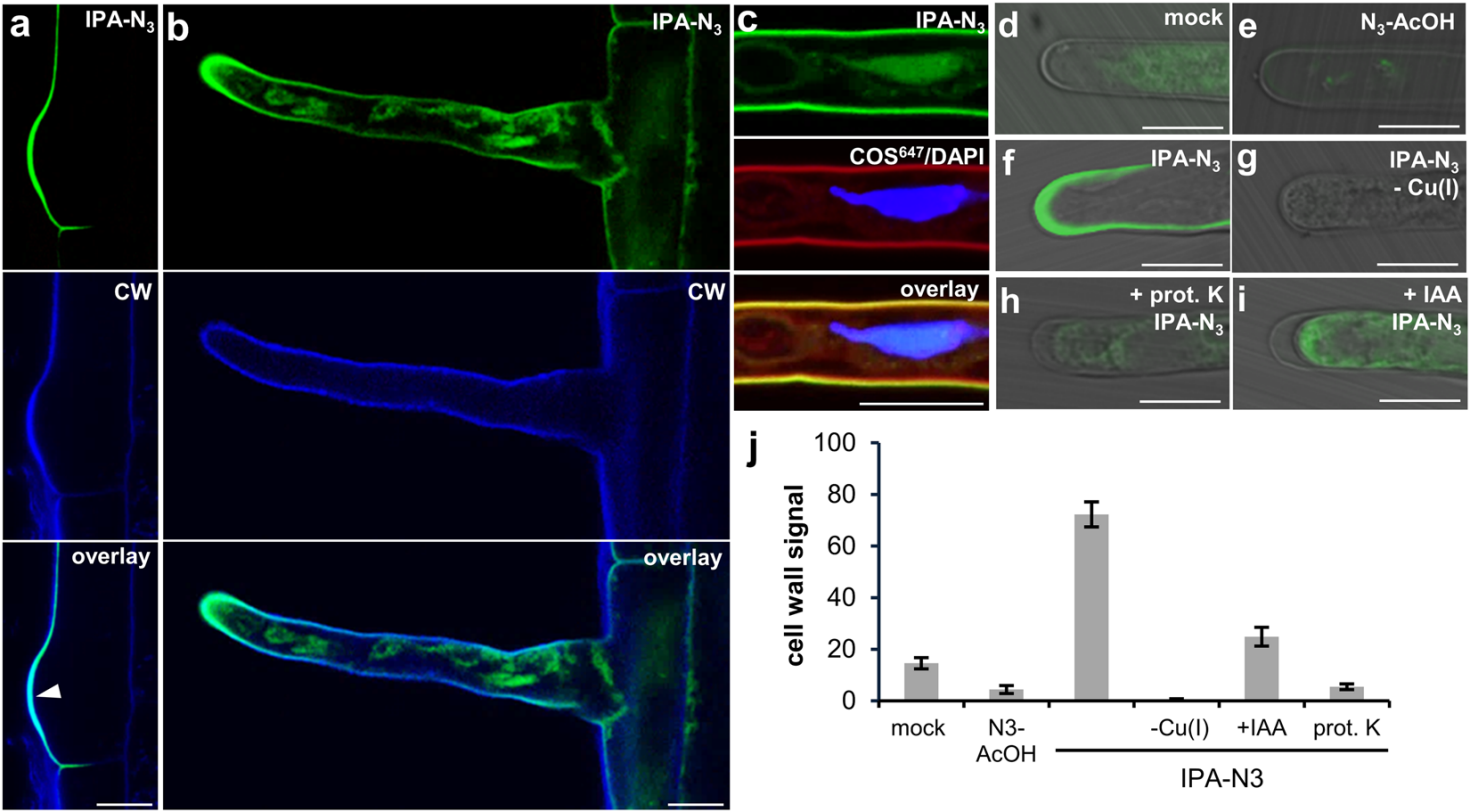
Localization of IPA-N_3_ in *Arabidopsis* root hairs. (**a**,**b**) The IPA-N_3_ localization in the outgrowing root hair (arrowheads) at the stage of (**a**) initiation and (**b**) elongation up to app. 100 μm in length. Upper panels: signal obtained after click reaction to alkyne functionalised Alexa Fluor 488. Middle panels: Counterstain with Calcofluor White (CW) marking cell wall. Bottom panels: overlay of the channels confirms the cell wall localization of IPA-N_3_. (**c**) The IPA-N_3_ localization (green channel) in the elongated root hair, co-stained by two cellular markers: COS^647^ (red channel) staining cell wall pectin components and DAPI (blue channel) staining nucleus. Overlay confirms the IPA-N_3_ localization in the cell wall. Some signal could be observed in the nucleus and a compartment surrounding the nucleus. (**d**–**i**) Controls for the IPA-N_3_ labelling in *Arabidopsis* root hairs. No visible cell wall labelling was observed in the case of seedlings incubated with (**d**) mock only or (**e**) control compound 2-azido acetic acid. (**f**) IPA-N_3_ labelling of root hair cell wall (10 μM, 1 h). (**g**) Labelling in the absence of copper catalyst, (**h**) when pre-treated with proteinase K, (**i**) or pre-incubated first with IAA (10 μM, 30 min). (**j**) Quantification of the cell wall signal (n = 5) from the labellings from (**d**–**i**). Scale bars = 10 μm.

The IPA-N_3_ signal could be observed in cell walls from the early stages of root hair formation (Fig. 5a,b). This was confirmed by co-localization with two independent cell wall markers^34,38^ (Fig. 5a–c) by plasmolysis of the cells (Supplementary Fig. S2a,b) and using another detection fluorophore - Cy3-alkyne (Fig. S2c). To determine the specificity of cell wall labelling, we also performed several additional control experiments (Fig. 5d–j) which again included depletion of the IPA-N_3_ signal by the pre-treatment of the seedlings with 10 μM IAA for 30 min. (Fig. 5i) or by simultaneously co-incubating with 10 μM IAA (Supplementary Fig. S2d). This strongly suggested the existence of competitive binding sites of IAA and IPA-N_3_ in the root hair cell wall.

### Conjugates of IAA or IPA to fluorophores do not bind to cell walls

We further characterised these binding sites using *in vitro* conjugates of IPA-N_3_ and IAA to fluorophores. The *in vitro* generated conjugate of IPA-N_3_ and Alexa Fluor 488 (Supplementary Fig. S2e,f) did not bind to the cell wall, which suggests that the presumed auxin binder in the cell wall does not tolerate larger substitutions on auxin, and may explain why the previously reported fluorescent conjugates of IAA did not label root hair cell walls^18^. The lack of binding of BOPA-N_3_ (Supplementary Fig. S2g,h) further indicated that these sites are not promiscuous towards just any aromatic ring-containing compounds.

**Figure 6:**
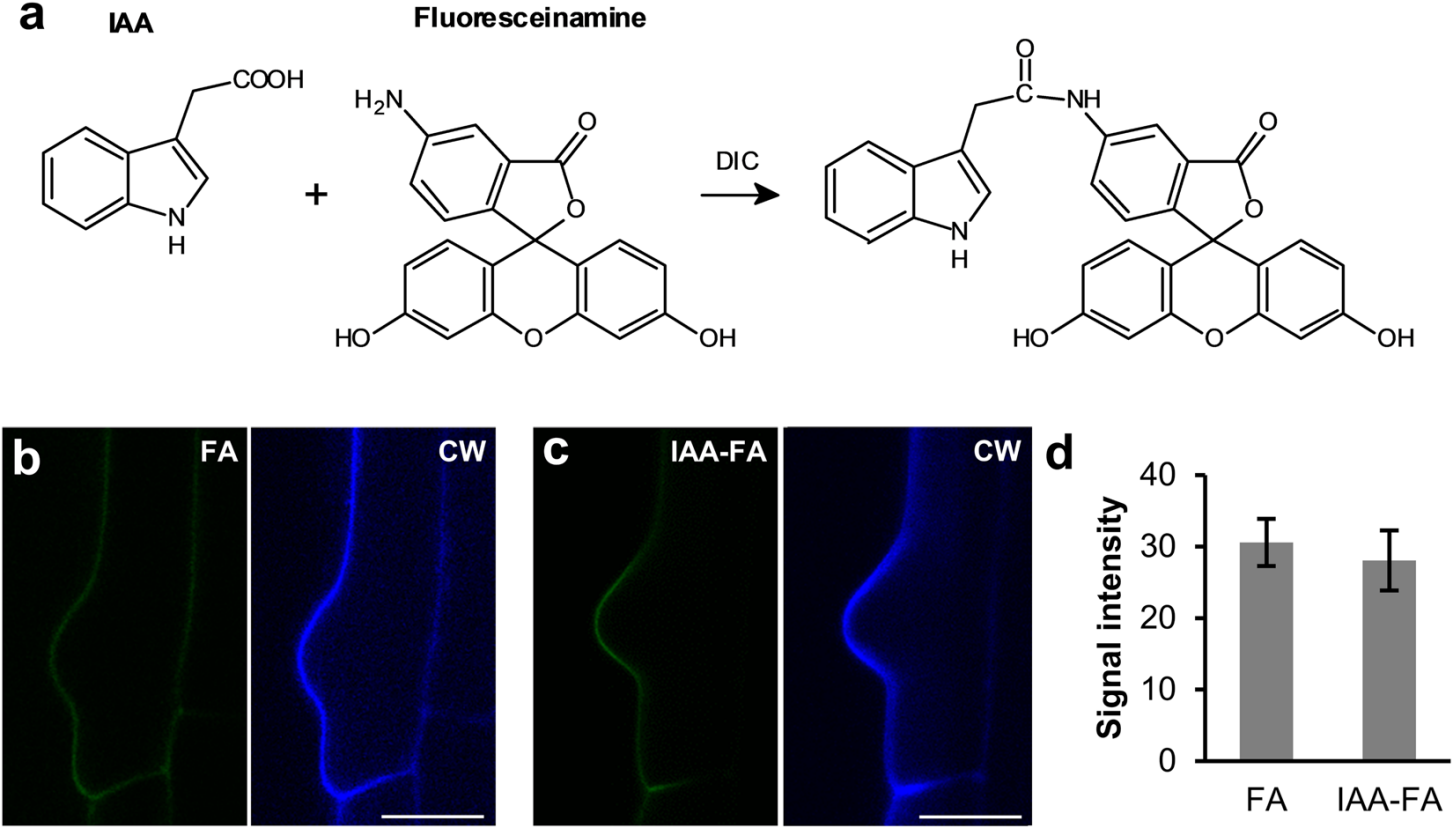
IAA conjugate to fluorophore does not bind to cell wall. (**a**) Scheme of the generation of the conjugate of IAA and fluoresceinamine (FA) using N,N’-diisopropylcarbodiimide (DIC) and amide formation. (**b**,**c**) *In vivo* labelling of *Arabidopsis* seedlings with 10 μM for 1 hour (**b**) using fluoresceinamine alone and (**c**) its conjugate to IAA (IAA-FA). Scan of the trichoblast with emerging root hair. Note only week signal in both labellings (CW, Calcofluor White). (**d**) Quantification of the cell wall signal confirms no differences between FA and IAA-FA labelling. Scale bars = 10 μm.

We further elaborated on this notion and generated a control compound in which the carboxy group of IAA was used for conjugation to fluoresceinamine (FA) via the formation of an amide bond (Fig. 6a and Supplementary Fig. S3). When compared to unconjugated FA, no specific cell wall signal could be observed in the elongation zone or root hairs (Fig. 6b–d). These experiments supported the validity of the click chemistry approach and suggested that the cell wall binding sites do not tolerate larger substitution on auxinic compounds and/or loss of free carboxy group.

### Anti-IAA antibody confirms presence of auxin epitopes in extracellular space

**Figure 7:**
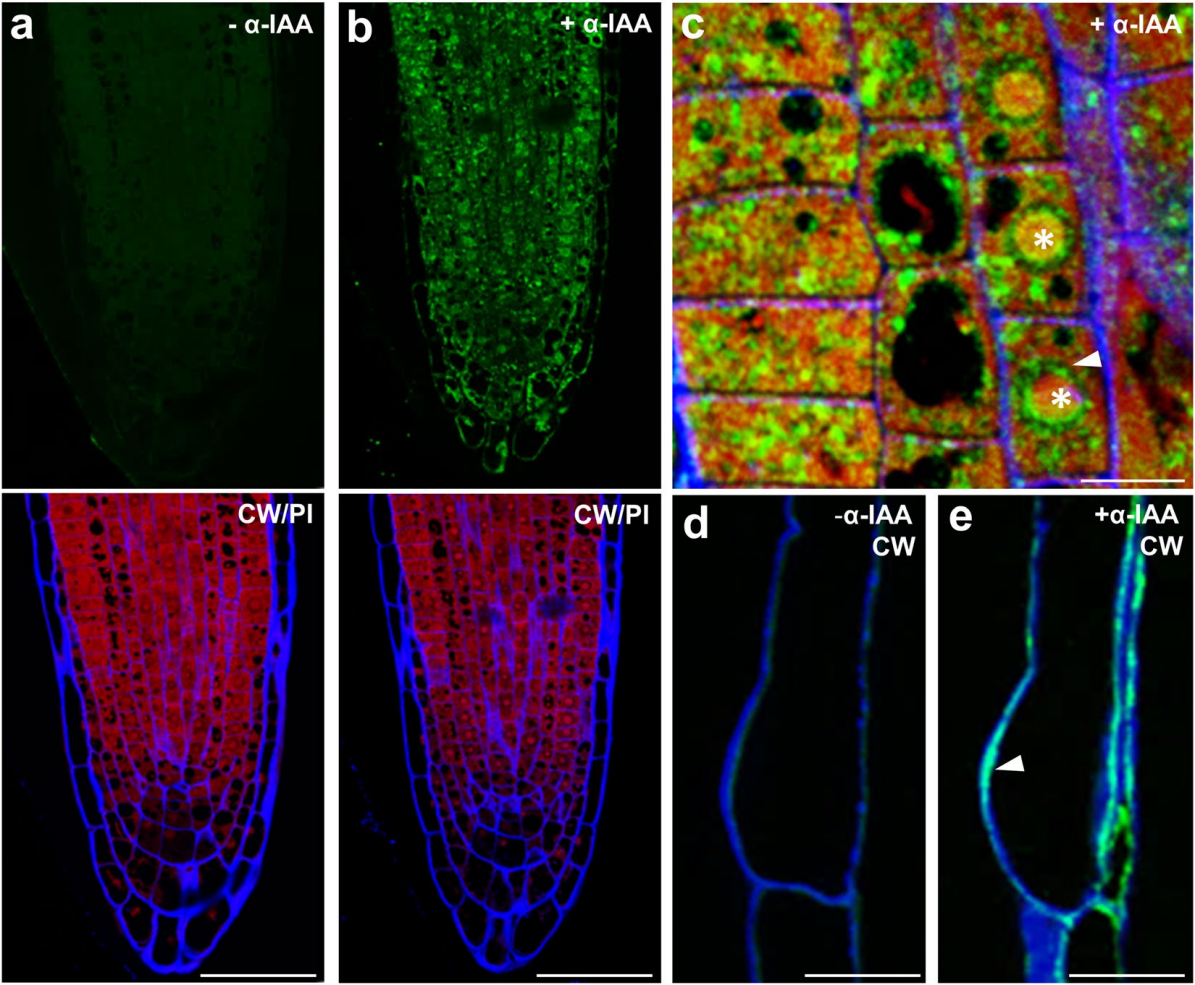
Immunolocalization of endogenous IAA epitopes in resin embedded *Arabidopsis* root. (**a**) The control sections stained with Alexa Fluor 488 -conjugated secondary antibody only (upper panel). (**b**) Detection of endogenous IAA epitopes using anti-IAA as primary antibody and Alexa Fluor 488-conjugated secondary antibody (upper panel). Bottom panels: counterstain of the same sections with propidium iodide marking nuclei (PI; red channel) and Calcofluor White marking cell walls (CW; blue channel). (**c**) Close-up of the root tip cells as overlay with both counterstains. Asterisks depict nuclear localisation and arrowhead endomembrane accumulation. Note the predominant intracellular localisation. In a few instances, observed cell wall localization of IAA epitopes might be due to intercellular polar auxin transport. (**d**,**e**) Localization of IAA using anti-IAA antibody in a trichoblast. (**d**) Control labelling, with no detected signal and (**e**) the cell wall accumulation of IAA at the tip of outgrowing root hair (arrowhead). Scale bars = 20 μm (**a**,**b**), 10 μm (**c**–**e**).

Previous experiments had raised an important question whether endogenously produced IAA is also targeted to root hair cell walls. To address this, we used an anti-IAA antibody for immunolocalization (Agrisera, AS09 445), generated using IAA conjugated to BSA via the C1 carboxyl group. The whole mount immunological detection of IAA resulted in only very faint staining in the root hair cell walls, which could be due to limited physical accessibility of the antibody to the epitope. To address this, we performed resin embedding and semi-thin sectioning of root tips. The IAA antibody labelling in the root meristem was exclusively intracellular, including nuclei (Fig. 7a–c). However, in trichoblasts, we could detect strong auxin accumulation in the cell wall at the site of outgrowing root hair (Fig. 7d,e). These observations supported the notion that our click chemistry approach indeed visualises auxin association with the cell walls with possible relevance to cell physiology.

### Cell wall-located auxin binding sites are distinct from ABP1

One of the potential candidates for auxin binding sites in the cell wall is the extensively studied AUXIN BINDING PROTEIN 1 (ABP1)^6,39^. ABP1 has been considered a crucial receptor involved in rapid non-transcriptionally mediated auxin responses^40–44^. However, the recently discovered viable full knock-out mutants of ABP1 questioned its importance in auxin signalling^45^. We tested IPA-N_3_ binding on the *abp1-c1* null mutant. Binding to the root hair cell walls was retained in the *abp1-c1* mutant (Supplementary Fig. S4), implying that ABP1 is probably not the sole provider of auxin binding sites in cell walls and that some of the other structures with affinity to auxin could be present in the apoplast. We explored the possibility that auxin associates with carbohydrates using defined carbohydrate microarrays populated with the most common cell wall components present in higher plants^46^. No IPA-N_3_ binding was detected to any polysaccharides or proteoglycans (Supplementary Fig. S5). Furthermore, treatment with proteinase K (Figs 4i and 5h) strongly diminished the fluorescence labelling *in situ*. Taken together, these data suggest the existence of additional secreted auxin binding proteins with unknown identity.

### IPA-N_3_ modulates cell expansion in pea

**Figure 8:**
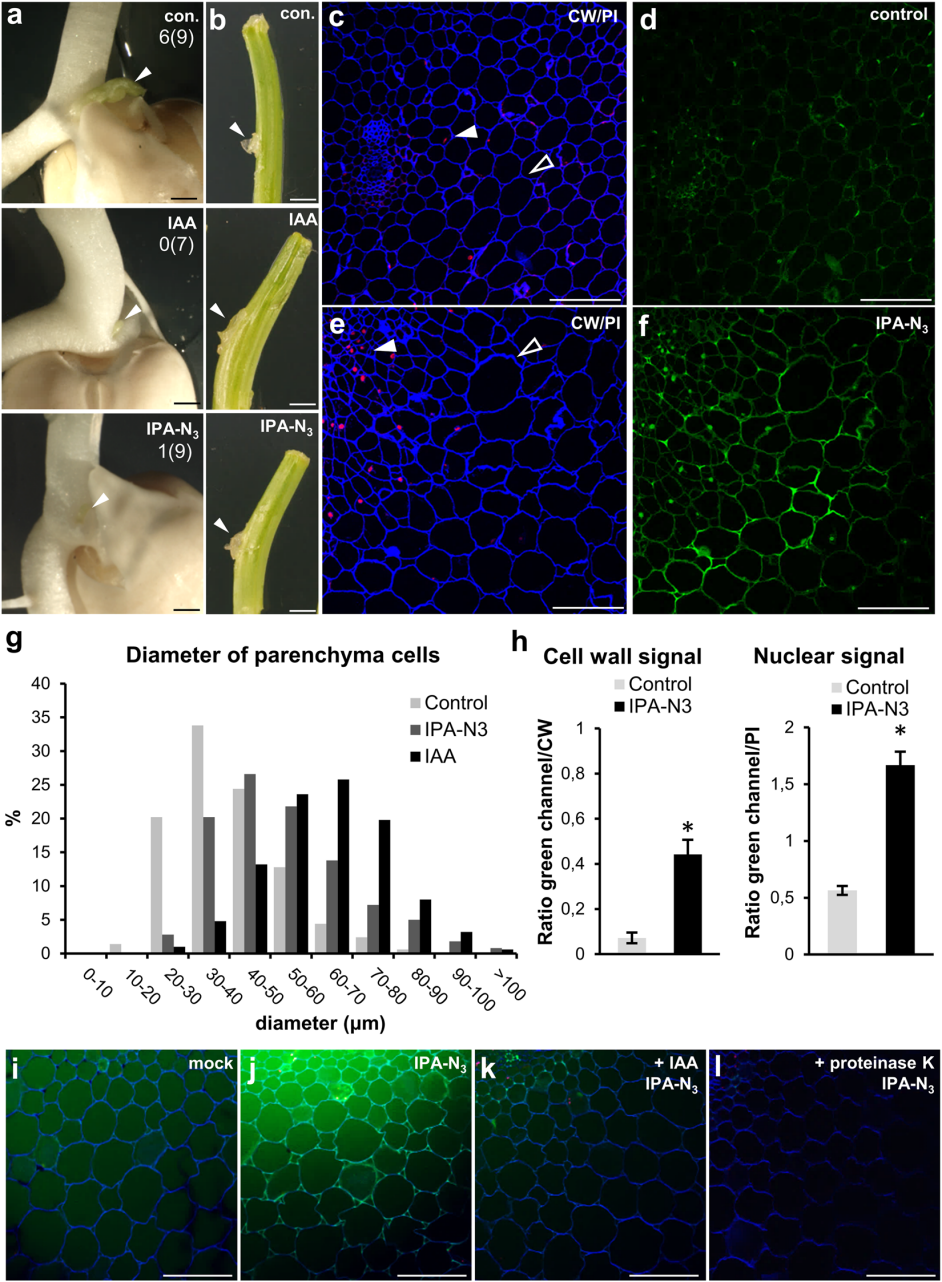
Physiological effects induced by IPA-N_3_ and its localization in pea epicotyls. (**a**,**b**) Effects on pea plants induced by application of lanolin paste containing IAA or IPA-N_3_ (30 mM). (**a**) When applied on the decapitated hypocotyl, both IAA and IPA-N_3_ inhibited the outgrowth of lateral buds (arrowheads). The numbers indicate the cases of the bud outgrowth from the total number of plants (in brackets). (**b**) Unlike lanolin mock treatment, lateral application of lanolin with IAA or IPA-N_3_ to the pea stem induced bending (arrowheads mark the site of application). The experiment was repeated twice with at least five stems treated for each compound. (**c**–**f**) Resin sections of the pea epicotyls 5 mm below the decapitation wound treated by (**c**,**d**) lanolin only and (**e**,**f**) lanolin containing 30 mM IPA-N_3_. (**c**,**e**) Staining with Calcofluor White marking cell walls (CW; blue channel) and propidium iodide marking nuclei (PI; red channel). Note the ectopic cell divisions (closed arrowhead) and cell swelling of parenchymatic cells (open arrowhead) in (**d**). (**d**,**f**) Click chemistry detection of IPA-N_3_ in the section reveals cell wall and nuclear localization. (**g**) Quantification of the diameter of the parenchymatic cells (three layers from the epidermis) in control, IAA and IPA-N_3_ treated epicotyls. 500 cells were measured for each treatment. (**h**) Quantification of the IPA-N_3_ nuclear and cell wall signal as the ratio between the green channel and respective cellular markers (propidium iodide and Calcofluor White) in the control and IPA-N_3_ treated sample. The mean of 20 measurements ± SE. *p < 0.01 (Student’s *t*-test). (**i**–**l**) Properties of the cell wall auxin binding sites in pea epicotyl parenchyma. (**i**) Labelling of mock-incubated control. (**j**) Incubation with 5 μM IPA-N_3_ for 15 min is sufficient for labelling of cell walls. Note that the cytoplasm is washed out due to the nature of vibratome sectioning. (**k**,**l**) The binding of IPA-N_3_ can be inhibited by pre-treatment of the sections with (**k**) IAA (10 μM, 15 min) and (**l**) by degradation of proteins using proteinase K. Scale bars = 5 mm (**a**,**b**), 300 μm (**c**–**f**,**i**–**l**).

Using pea (*Pisum sativum*), a classical model for auxin studies^7^, and IPA-N_3_ we investigated the relationship between auxin in the cell wall, its transport, and regulation of elongation. In contrast to *Arabidopsis* seedlings, pea plants are amenable to modulation of auxin transport by the local application of lanolin paste containing auxin. The lateral application of IAA and IPA-N_3_ to pea stems induced bending and inhibited outgrowth of lateral buds when applied on the top of decapitated epicotyls (Fig. 8a,b). This shows that IPA-N_3_ is a transportable auxin which can also alter the rate of cell elongation. To study the movement of IPA-N_3_ through hypocotyls and its distribution, paste containing IPA-N_3_ was applied to decapitated seedlings, which were then allowed to grow for 24 h. The region 5 mm below the wound was analysed by sectioning resin embedded material followed by click-labelling (Fig. 8c–f). In contrast to control, IAA and IPA-N_3_ induced cell swelling and massive cell divisions (Fig. 8c,e,g and Supplementary Fig. S6a–c). These two effects could be linked to the IPA-N_3_ labelling visible in both the extracellular region and the nuclei (Fig. 8d,f,h).

To exclude the possibility that the extracellular IPA-N_3_ accumulation is due to cell-to-cell transport or diffusion, we analysed the properties of auxin binding sites on fresh vibratome-generated sections through epicotyls. A short (15 min) application of IPA-N_3_ to sections was sufficient for cell wall labelling, using both a fluorescent tag and biotin with exclusive binding to parenchyma cells (Fig. 8i,j and Supplementary Fig. S6d,e). Again the intensity of the fluorescent signal was partly inhibited by pre-incubation with IAA, and completely inhibited by the removal of proteins using proteinase K pre-treatment (Fig. 8k,l). These data further confirm that IPA-N_3_ binds to cell wall-located proteinaceous auxin binding sites which might have a relation to cell wall extendibility during elongation.

### Some elongating cells lack IPA-N_3_ binding to cell walls

Previous experiments conclusively suggested that there is a strong relationship between auxin binding sites in the cell wall and cell expansion/elongation. We investigated this further on different types of elongating cells. The pollen tube is another example of an elongating (tip-grown) cell structure and the role of auxin during pollen tube growth has also been suggested^47^. However, in this biological system we did not observe IPA-N_3_ cell wall binding even at the rapid growth stage 4 h after germination (Supplementary Fig. S7a). The lack of auxin cell wall binding sites was also apparent for elongated protonema cells of the moss *Physcomitrella patens* (Supplementary Fig. S7b), known already to possess complex auxin regulation (synthesis, transport and signalling) similar to higher land plants^48^. These observations suggest that the presence of auxin binding sites *in muro* is restricted to certain elongating cells expressing corresponding binding proteins.

## Discussion

We introduced IPA-N_3_ as a novel click chemistry-compatible auxinic compound which can serve as an auxin tracer for some applications. Although several proteins have been hypothesized to be auxin binding, ABP1 has long been considered the most important and has been extensively studied^6,39,49^. Our results contribute to the debate providing evidence for putative secreted proteinaceous auxinic binding sites that are distinct from ABP1. The long-time standing ‘acid growth theory’ proposes that auxin promotes expansion by regulating pH-dependent rearrangement of cell wall components^7,8^. Although this auxin activity is most likely to be regulated via the nuclear SCF(TIR1/AFB) pathway^13^, other non-genomic effects of auxin including the one on microtubules have been suggested^50^, but widely disputed^51,52^.

Observed accumulation of IPA-N_3_ and detection of IAA epitopes in apoplast exclusively in cells undergoing changes in their size, intriguingly stimulates the notion of some direct auxin-triggered growth control of the structural dynamics of cell walls. We speculate that this putative pathway(s) is (are) not necessarily elongation-promoting or directly linked to acid growth, but may play a supporting role in the modulation of some cell wall remodelling via cell wall-bound enzymes or regulating cellular auxin homeostasis. Although this remains a novel hypothesis for further testing, we believe that the approach presented for detecting auxinic compounds in the cell wall can be an important asset for unravelling some enigmatic aspects of plant growth and development.

Unfortunately, the presented method has some limitations in terms of penetration into deeper tissues, for example in *Arabidopsis* root tips. Although IPA-N_3_ was able to trigger auxin response in deeper layers of the root tip (Fig. 3), it was detected mainly in the outer cell layers of the root tip in both whole mount and resin sections, suggesting problems with fixation. In this case it might not fully reflect the auxin accumulation sites as in the case with fluorescent analogues. If future optimization of the method overcomes these shortcomings, it will greatly benefit from the general advantages of click chemistry. Click chemistry-ready auxin analogues can be applied in whole mount imaging without using any harsh treatment (e.g. the partial dissolution of cell walls using driselase), as is often the case in plant immunohistology. Other advantages are fixability, compatibility with other cytological staining methods, and high versatility in the choice of tags which can be used for conjugation.

## Methods

### Plant material and growth conditions

*Arabidopsis thaliana* (L.) Heynh. seedlings were grown on vertical plates with Murashige-Skoog (MS) medium supplemented with 1% sucrose in the growth chamber at 21 °C and 16 h photoperiod for three days. The adult plants were grown in the glasshouse. The following genotypes have been used: Col-0, *DR5rev::GFP* ^16^, R2D2^33^, *abp1-c1* ^45^, *pin1-1* ^32^. Pea seeds (*Pisum sativum* (L.) var Norli) were sterilized by full strength hypochlorite solution for five min, washed and soaked in water for two hours and left to germinate in a wet chamber for three days in the dark. Pollen germination was performed as described^53^, on cellophane-covered pads for 4 h at 21 °C. Cellophane support with pollen was removed and incubated in small sieves in solutions in a 24-well plate. *Physcomitrella patens* (Hedw.) ecotype Gransden was grown on solid Phy B media under the same growth conditions as *Arabidopsis*.

### Liquid chromatography–mass spectrometry (LC-MS) analyses

LC–MS was performed using an Agilent 1100 Series LC (Agilent Technologies) coupled to a Bruker HCT-Ultra ion trap mass spectrometer (Bruker Daltonics), as described^54^. The LC–MS data was analysed using Bruker-DataAnalysis 4.0 (Bruker Daltonics). The standards were run diluted in water solutions. The pea homogenate was produced by grinding 500 mg of cut fresh pea epicotyls using a pestle following followed by 10 min of centrifugation at 10 000 *g*. The undiluted supernatant was used for *in vitro* tests of metabolic stability.

### Click chemistry

All reagents have been purchased from Sigma-Aldrich or Invitrogen. The stock solutions of auxin analogues were made in ethanol, or, in the case of BOPA-N_3,_ in DMSO at 10 mM concentration. The plant material was incubated with the mock or IPA-N_3_ (Sigma; CAS: 79410-36-9) in the liquid MS media, washed twice, fixed with 2% 1-ethyl-3-(3-dimethylaminopropyl)carbodiimide (EDAC) in PBS for 15 min, then 30 min in 4% PFA washed with PBS, permeabilized by 1% DMSO/0.3% NP40 in PBS for 15 min and washed with PBS. After blocking with 3% BSA, the click chemistry was performed according to the manufacturer’s instructions (Invitrogen), using the Alexa Fluor 488 5-Carboxamido-(Propargyl) (Invitrogen; A10267), Biotin-PEG4-alkyne (Aldrich 764213) or Cy3-alkyne (Aldrich 777331) at 3 μM concentration and Cu(I) in the reaction mixture for 30 min. Samples were washed with 3% BSA, counterstained by Calcofluor White, propidium iodide, COS^647^ (ref.^38^) at 1:1000 dilution in MES buffer pH 5.7 or DAPI, for 10 min, washed with PBS, mounted in CitiFluor (Agar Scientific) and immediately observed. In the case of the biotin-PEG4-alkyne labelling, the samples were incubated with a streptavidin-alkaline phosphatase conjugate (Sigma; S2890) at a 1:2000 dilution for 30 min, washed with water and incubated in the development solution containing NBP/BCIP substrates in alkaline phosphatase buffer and observed using a light microscope.

### Generation and analysis of IAA-fluoresceinamine

10 mg of IAA were dissolved in 500 μl of DMF together with 50 mg of fluoresceinamine (isomer I) and 90 µl of N,N′-diisopropylcarbodiimide (DIC) were added (both from Sigma). The reaction was left to proceed overnight at r.t. with shaking at 1400 rpm in darkness. The reaction was followed by TLC using dichloromethane:methanol (7:3) as an elution solvent and staining with the Ehrlich reagent (1 g of 4-(dimethylamino)-benzaldehyde (DMAB) dissolved in 25 ml of 96% EtOH and 25 ml of concentrated HCl). Analyses of the product were performed by UHPLC-MS on a RSLC Dionex Ultimate 3000 (Thermo) coupled to a QTOF Impact HD (Bruker) on a Kinetex 2.6 µm EVO 100 Å C18 column (50 × 2.1 mm, Phenomenex). The calculated molecular weight of the product [M + H]+ was 505.1394 amu and the determined molecular weight was 505.1405 amu (mass accuracy 2.17 ppm).

### Resin sections of pea epicotyls and *Arabidopsis* roots

Three-day-old *Arabidopsis* seedlings and two mm thick pea epicotyl slices were fixed with 2% EDAC for 15 min and 4% PFA in PBS for 30 min and embedded in LR White resin (Sigma) using methanol series for dehydration. Polymerization was performed in gelatine capsules at 60 °C overnight. The 1 μm-thick sections were prepared using a glass knife on a Leica Ultramicrotome EM-UC7. The sections were adhered to the SuperFrost slides and were directly click-labelled or used for auxin immunolocalization: sections were blocked by 3% BSA for 30 min and probed with the anti-IAA antibody (Agrisera, AS09 445) at the dilution of 1:100 for 2 h, washed and probed with the secondary anti-rabbit antibody conjugated to Alexa Fluor 488 at a 1:300 dilution for 1 h, washed and counterstained by Calcofluor White (0.1 mg/ml) and propidium iodide (10 μM) and mounted in CitiFluor.

### Auxin analogues sensitivity test

Three-day-old seedlings grown on the vertical plate were transferred to 1 ml of liquid MS medium in a 24-well plate supplemented with tested compounds and grown for 48 h in the growth chamber. The seedlings were stretched out on the fresh plate and scanned by a flatbed scanner; and the length of the roots and hypocotyls was measured using ImageJ software (http://rsb.info.nih.gov/ij/index.html). The organ initiation was performed on four-week-old *pin1* plants as described^31^, using 1 mM concentration of IAA and IPA-N_3_ in lanolin. The apices were photographed four days after micromanipulation. For the apical dominance tests, three-day-old seedlings growing in the dark were decapitated and the lanoline paste containing auxins was applied (30 mM) on the wound and left to grow for the next three days in the dark. For the pea stem elongation assay, young internodes on the four-week-old plants app. 2 cm long were decapitated and the wound was sealed by lanoline. The lanoline containing auxins (30 mM) was applied laterally in the middle of the stems and observed after two days.

### Quantitative RT-PCR

Quantitative RT-PCR was carried out as described previously^55^, using a LightCycler 480 (Roche) with LightCycler 480 SYBR Green master mix (Roche) and the manufacturer’s qRT-PCR program recommendations. Four technical repeats were carried out to assess the gene expression levels. Gene expression was normalised to UBQ10^55^ and to EIF^56^ genes (Supplementary Table S1), giving results of similar statistical significance; the representative values normalised to UBQ10 are shown. Equal variances of datasets for ANOVA were verified by the Levene test, and the Kruskal–Wallis nonparametric test was performed simultaneously with ANOVA. Data were evaluated with NCSS 2007.

### Confocal Laser Scanning Microscopy (CLSM) and light microscopy

The water- or CitiFluor-mounted samples were scanned on Leica SP5 confocal microscope equipped with a UV diode (405 nm), Argon (488 nm) and Argon-Krypton (647 nm) lasers. The light microscopy was undertaken using an Olympus BX41 microscope. The pictures were processed with GIMP2 software for cropping, brightness and contrast improvement. Pictures for comparison were treated equally.

### Defined carbohydrate microarrays

The carbohydrate microarrays have been prepared as described^46^, containing 23 defined polysaccharides and proteoglycans using Arrayjet microarray printers in four dilutions. The arrays were blocked with 5% milk in PBS incubated with IPA-N_3_ for 1 h washed and reacted to the Alexa Fluor 488-alkyne. The arrays were washed, dried and scanned using a GenePix 4400 A microarray scanner.

## Electronic supplementary material

Supplementary Information
