# Supplementary material for: Click chemistry-based tracking reveals putative cell wall-located auxin binding sites in expanding cells
Source: Sci Rep. 2017 Nov 22;7:15988. doi: 10.1038/s41598-017-16281-w (PMC5700113; doi:10.1038/s41598-017-16281-w)
Supplement: Supplementary file 1 — Supplementary Information [file 41598_2017_16281_MOESM1_ESM.pdf]

Supplementary Information

Click chemistry-based tracking reveals putative cell wall-located auxin binding sites in expanding cells

Jozef Mravec, Stjepan K. Kračun, Elena Zemlyanskaya, Maja G. Rydahl, Xiaoyuan Guo, Martina Pičmanová, Kasper Kildegaard Sørensen, Kamil Růžička and William G. T. Willats

Tab. S1: List of primers used
Fig. S1: Analysis of the purity and the metabolic stability of IPA-N_3
Fig. S2: Additional controls for click chemistry labelling in Arabidopsis
Fig. S3: Analysis of the IAA-FA conjugate with MS
Fig. S4: Analysis of IPA-N_3 binding in *abp1* mutant
Fig. S5: Analysis of IPA-N_3 affinity to carbohydrates and proteoglycans using defined carbohydrate microarrays
Fig. S6: IPA-N_3 induces cell swelling and ectopic cell divisions and binds to pea epicotyl parenchyma cell walls.
Fig. S7: IPA-N_3 does not bind to cell walls of two types of elongating cells.

Table S1

Name	Sequence
qUBQ10_F	CACACTCCACTTGGTCTTGCGT
qUBQ10_R	TGGTCTTTCCGGTGAGAGTCTTCA
qEIF4A_F	ACGGAGACATGGACCAGAAC
qEIF4A_R	GCTGAGTTGGGAGATCGAAG
SHY2_F	GGGCAAGATCTATGTTCATTGG
SHY2_R	ACCTTTTGCCCTGTTTCTGA
IAA2_F	GAAGAATCTACACCTCCTACCAA
IAA2_R	CACGTAGCTCACACTGTTGTTG
IAA5_F	AGATGTTCCTTGGGAAATGTTCCT
IAA5_R	GCAGTAGCTTATAAGAACCGCCT
IAA10_F	CTTCTCCTGTAACAAGATCCAACAC
IAA10_R	CTCTACTTACCTACTCCAGCTCCA

Fig. S1

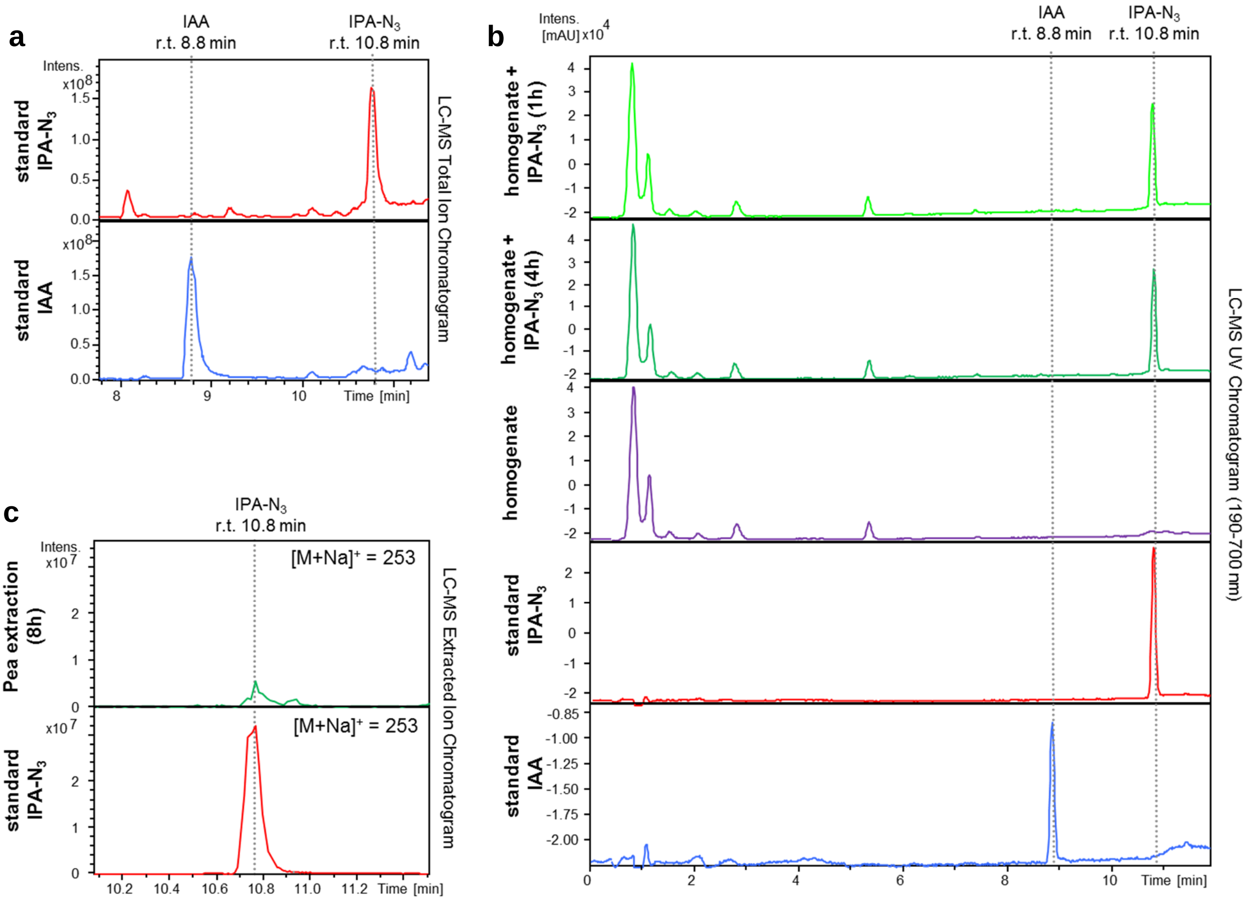

Fig. S1: Analysis of the purity and the metabolic stability of IPA-N_3

(**a**) LC-MS analyses of the IAA and IPA-N_3 standards diluted in water at 1 mM concentration. No IAA was detected in the commercial preparation of IPA-N_3. (r.t. – retention time). (**b**) *In vitro* test for the metabolic stability of IPA-N_3. The 20 µl of pea homogenate from epicotyls was mixed with 200 µl of 500 µM IPA-N_3 solution in water and incubated for 1 and 4 h respectively at room temperature. No significant degradation of IPA-N_3 or any presence of IAA after 4 h of incubation was detected. (**c**) *In vivo* test for the IPA-N_3 stability: 500 µl of water solution of 1 mM IPA-N_3 was incubated with 500 mg of 2-5 mm-thick epicotyls slices infiltrated for 30 min by vacuum and incubated for 8 h. The epicotyls were washed twice and IPA-N_3 was extracted by methanol and analysed by LC-MS. We calculated that approx. 1-2 % of IPA-N_3 from the initial amount in the solution added could be extracted from the washed epicotyls after 8 h.

Fig. S2

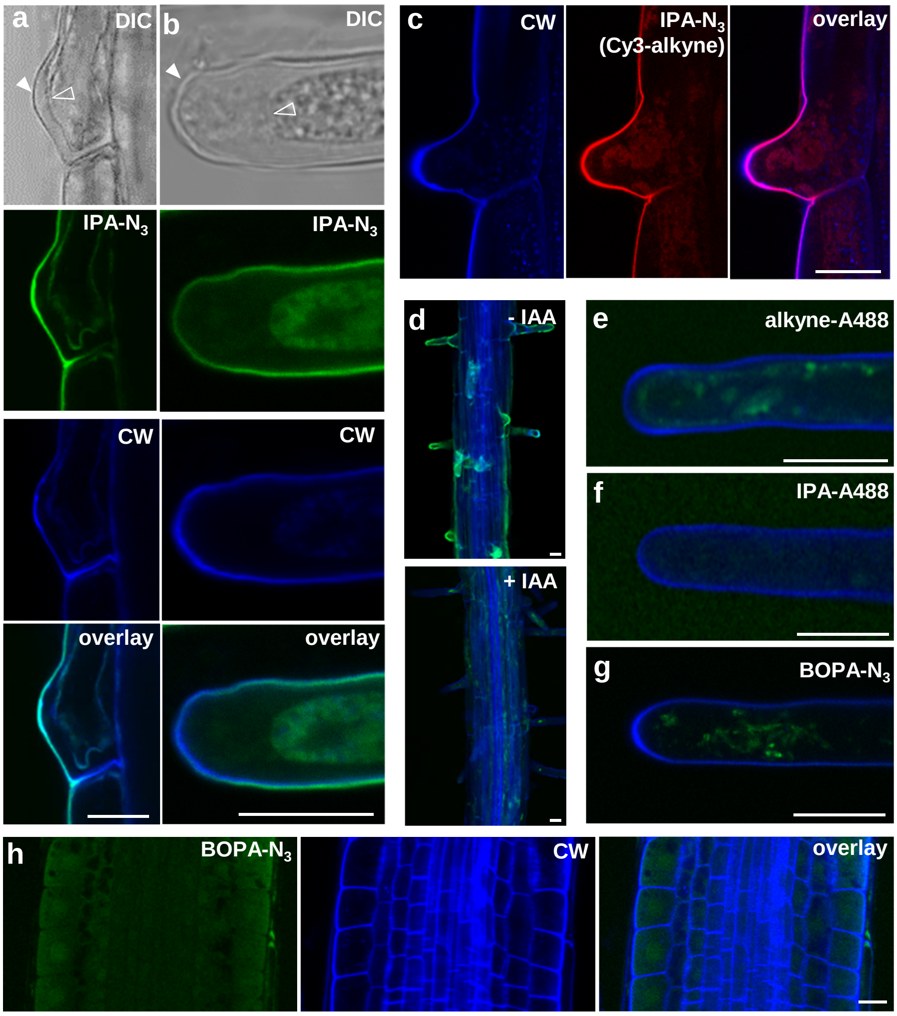

Fig. S2: Additional controls for click chemistry labelling in *Arabidopsis*
(**a**) Plasmolysis and co-localisation with Calcofluor White (CW) confirms cell wall localisation of IPA-N_3 signal in (**a**) trichoblasts with emerging root hair and (**b**) elongated root hair. Closed arrowheads mark the cell wall and open arrowheads shrank cytoplasm (DIC, differential interference contrast). (**c**) The IPA-N_3 can be traced to cell wall also using Cy3-alkyne fluorophore. (**d**) The binding of IPA-N_3 to root hairs and root epidermal cells could be inhibited by co-incubation (1 hour) with 10 µM IAA (**e-h**) Analyses of root hair localisation of three control compounds. Scans of root hairs of *Arabidopsis* roots incubated for 30 min with (**e**) alkyne derivative of Alexa Fluor 488 or (**f**) Alexa Fluor 488 clicked reacted *in vitro* to the 10 µM IPA-N_3 and then used for the labelling. (**g,h**) Localisation of a control compound 2-azido-3-benzyloxypropionic acid (BOPA-N_3) in root hairs (**g**) and root epidermal cells (**h**) using click chemistry procedure as described for IPA-N_3 (10 µM, 1 h incubation). In neither of the two cases any cell wall signal was observed. Scale bars = 10 µm.

Fig. S3

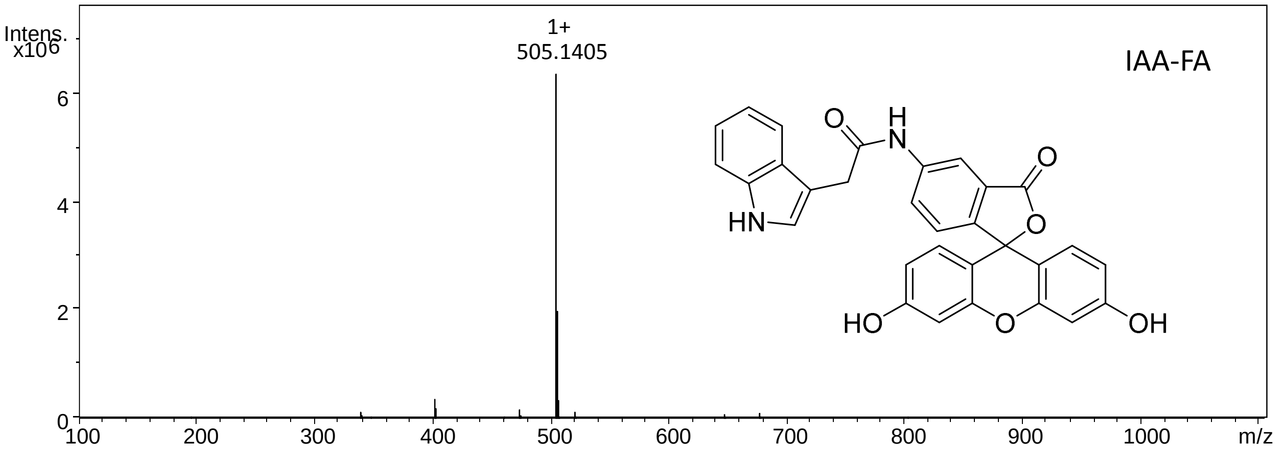

Fig S3. MS analysis of the IAA-fluoresceinamine (IAA-FA)
Chemical Formula: $C_{30}H_{20}N_2O_6$; Calculated Mass: 504,1321; found: $[M+H]^+$ 505.1405; Mass accuracy: 2.17 ppm

Fig. S4

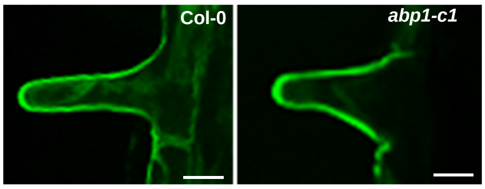

Fig. S4 Analysis of IPA-N_3 binding in *abp1* knock out mutant The IPA-N_3 cell wall labelling (10 µM) was also observed in null *abp1-c1* mutant. Scale bars = 10 µm.

Fig. S5

a

Pectins

Lime pectin DE: 0% (E0)
Lemon pectin
Apple pectin
CP Kelco Pectin
Esterified citrus pectin

RG I related

Sugar beet Arabinan
Linear Arabinan
Pectic galactan,
RGI (soy bean)
RGI (potato)

Hemicelluloses and proteoglycans

Mannan (ivory nut)
Galactomannan (carob)
Glucomannan (konjac)
Xylan (birch)
Arabinoxylan (wheat)
Xyloglucan Tamarind seed
Lichenan, β-glucan (1→3),(1→4)-β-D-glucan
β-glucan (Yeast), (1→6),(1→3)-β-D-glucan
β-glucan, (1→3),(1→4)-β-D-glucan
Pachyman (1→3)-β-D-glucan
Arabinogalactan, type II (AGP)
Locust bean gum, Galactomannan rich gum
Gum arabic

Oligosaccharides

α-(1-5)-L-Arabinooctaose (furanose ring)
D-Galactose
β-(1-4)-D-Mannohexaose
Xyloglucan heptamer, XXXG~OH
Xyloglucan oligosacc. XXXGXXX(G), reduced
β-(1-4)-D-Xylohexaose (pyranose form)
β-(1-3),(1-4)-D-Glucotetraose [G4G3G4G~OH]
β-(1-3)-D-Gluco, Laminarihexaose
Maltohexaose, α-(1-4)-D-Glucohexaose
hexa acetyl-chitopentaose
Cellohexaose, β-(1-4)-D-Glucohexaose
β-D-lactose, galactosyl-β-(1→4)-D-glucose

b **c**

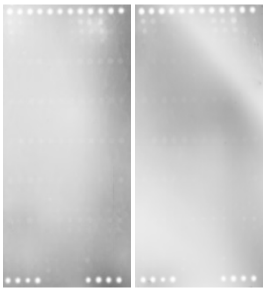

Fig. S5: Analysis of IPA-N_3 affinity to carbohydrates and proteoglycans using defined carbohydrate microarrays
(**a**) The list of poly-, oligosaccharides and proteoglycans present on the used microarrays.
(**b,c**) The fluorescent scans of the microarrays after IPA-N_3 incubation and the click reaction.
(**b**) Control microarray and (**c**) microarray incubated with IPA-N_3. In both cases no specific binding to any cell wall component could be observed. The white spots are printed ink margins.

Fig. S6

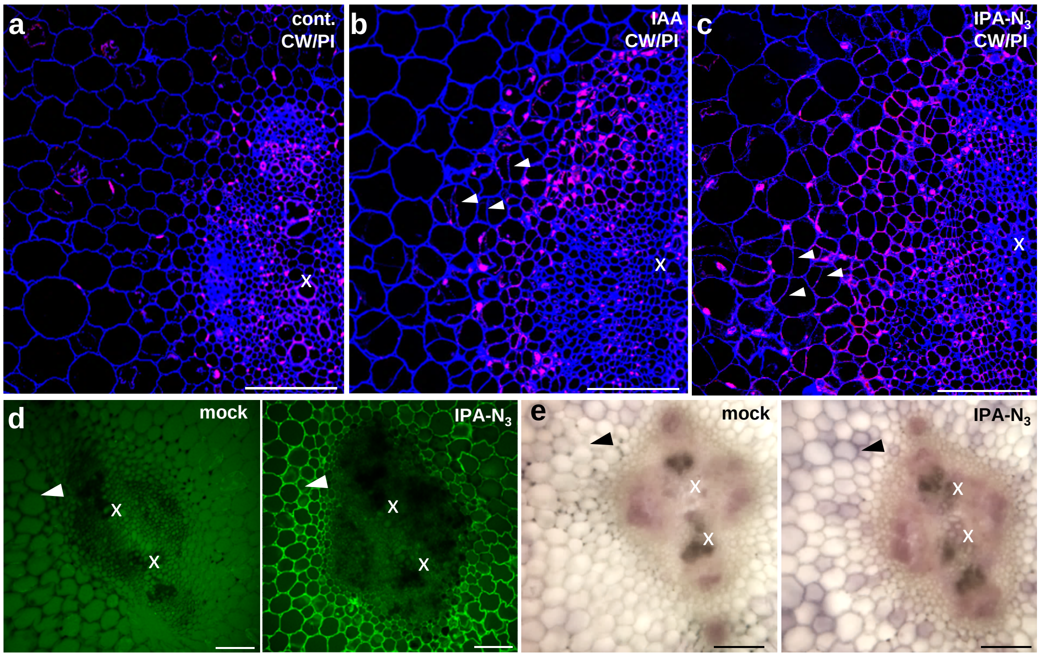

Fig. S6: IPA-N_3 induces cell swelling and cell divisions and binds to pea epicotyl parenchyma cell walls.

(**a-c**) The morphology of the epicotyls below the wound sealed with (**a**) lanoline only, (**b**) IAA and (**c**) IPA-N_3. Both IAA and IPA-N_3 induced cell swelling and cell divisions (note the number of freshly made septa, indicated with arrowheads). Staining with Calcofluor White marking cell walls (CW; blue channel) and propidium iodide marking nuclei (PI; red channel). (**d,e**) Binding of IPA-N_3 to the fresh sections of the pea hypocotyls using click labelling with (**d**) Alexa Fluor 488-alkyne, or (**e**) biotin-PEG4-alkyne subsequently detected by streptavidin-alkaline phosphatase. Note the specific binding to the parenchyma cells (arrowheads) in the case of both detection methods. The sclerenchyma cells and vascular tissues possibly exhibit endogenous phosphatase activity and show brown background signal also in the control. Nuclei cannot be seen, as they are usually disrupted during sectioning of the fresh material. The position of the xylem vessels is marked by x. Scale bars =100 µm.

Fig. S7

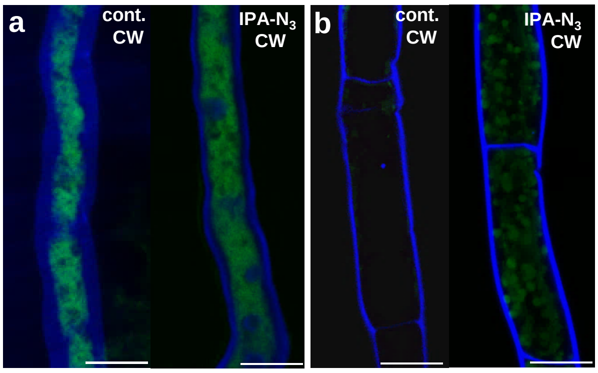

Fig. S7: IPA-N_3 does not bind to cell walls of two types of elongating cells. (**a**) Labelling of *Arabidopsis* pollen tube and (**b**) *Physcomitrella patens* protonema cells after 15 min incubation in 5 μM IPA-N_3. No cell wall binding could be observed. Calcofluor White (CW, blue channel) marks cell walls. Scale bars = 1 μm.
